# Unexpected early pregnancy during myomectomy followed by successful term delivery: A case report

**DOI:** 10.18502/ijrm.v22i8.17242

**Published:** 2024-10-14

**Authors:** Nasrin Saharkhiz, Mitra Nemati ., Nazanin Hajizadeh, Hajar Abbasi

**Affiliations:** Preventative Gynecology Research Center, Shahid Beheshti University of Medical Sciences, Tehran, Iran.

**Keywords:** Leiomyoma, Pregnancy, Uterine myomectomy.

## Abstract

**Background:**

Leiomyoma, also known as uterine fibroid, is a non-malignant tumor originating from the uterus's smooth muscles. It is the most common benign tumor in the female genital tract, exhibiting varying size and form that can distort the uterus's shape. Uterine fibroids affect 2.7–10.7% of pregnant women and can lead to increased risks during pregnancy, such as miscarriage, placental abruption, preterm labor, and fetal malpresentation. Myomectomy is a surgical intervention for uterine fibroids, but it has drawbacks, including hemorrhage, fever, infection, ureter ligation, adhesive disorders, and unplanned hysterectomy.

**Case Presentation:**

In this case report, we present a 32-yr-old woman with a large leiomyoma who underwent laparotomy myomectomy due to abdominal pain and heavy menstrual bleeding. 4 wk after the myomectomy, she was referred to the Obstetric Clinic of Ayatollah Taleghani hospital, Tehran, Iran complaining of delayed menstruation. Her beta-human chorionic gonadotropin test was positive. Ultrasound revealed a fetus with an estimated gestational age of 6 wk and 4 days. The pregnancy continued with no complications. At 38 wk of gestation, she underwent a cesarean section and delivered a healthy newborn.

**Conclusion:**

This case report supports previous publications that have demonstrated the safety of myomectomy during pregnancy.

## 1. Introduction

Leiomyoma, commonly known as uterine fibroid, is a non-malignant tumor that originates from the smooth muscles of the uterus. Uterine fibroids are the most common benign tumors of the female genital tract, with a broad spectrum of size and form that can distort the shape of the uterus (1). Clinical implications of uterine fibroid include abnormal uterine bleeding, bulk-related symptoms (hydronephrosis, adjacent organ pressure, and pain), and fertility issues. The prevalence of leiomyoma varies between 2.7–10.7% during pregnancy (2), which is positively associated with increasing maternal age (3). During pregnancy, uterine fibroids can be followed by an increased risk of miscarriage, placental abruption, preterm labor, and fetal malpresentation (1). Although leiomyomas usually remain asymptomatic during pregnancy, they may manifest abdominal pain, first-trimester bleeding, abnormal fetal growth, and labor dysfunction (4).

Management of uterine leiomyomas depends on various factors including the size, number, and location of the uterine fibroids, and the severity of symptoms. Usually, asymptomatic fibroids require no surgical procedures. However, certain situations may necessitate treatments including medications, high-intensity focused ultrasound, embolization, and myomectomy (5). Management of uterine fibroids in pregnant women could be challenging. Obstetrics and gynecology specialists prefer to treat uterine fibroids during pregnancy with expectant management. Nevertheless, in rare situations, surgical interventions like myomectomy may be required (4, 5). Uterus rupture can occur as a consequence of pregnancy after myomectomy and its incidence depends on different factors such as features of the leiomyoma and suture technique applied during myomectomy. There are controversies about the risk of uterus rupture and its complications in pregnant cases undergoing myomectomy (6).

Herein we presented a case of unexpected pregnancy that underwent myomectomy followed by successful term delivery.

## 2. Case Presentation

A 32-yr-old woman with a history of 2-term vaginal deliveries (gravid 2, para 2, and live 2) and insertion of copper intrauterine device, referred to Imam Hossein hospital clinic, Tehran, Iran suffered from heavy menstrual bleeding and pelvic pain during the preceding 6 months. Menstrual cycles were regular, with 2–3 days of heavy bleeding (soaking 6–7 pads per day) followed by moderate to severe bleeding for up to 7–8 days. She also reported mild dysmenorrhea in this period. On physical examination, she had stable vital signs. On the abdominal palpation a firm, irregular, non-tender mobile abdominopelvic mass was explored, resembling the size of a 14 wk pregnancy. On spaculum and bimanual examination, the cervix and adnexa were normal. The complete blood count revealed a hemoglobin concentration of 10.5 g/dl and a hematocrit level of 30%. Other laboratory tests were normal. On ultrasound assessment, the size of the uterus was 68 × 58 mm; a heterogeneous 71 × 69 cm mass was seen involving the anterior uterine wall on the uterine body portion with anechoic areas and endometrial compression. Bilateral ovaries were normal, and no free fluid was observed in the pelvic cavity. Due to the severity of symptoms and sonographic findings, myomectomy was considered for the treatment. After explaining the probable complications of myomectomy such as uterine rupture in a future pregnancy and the possibility of needing a hysterectomy during myomectomy, an informed consent was obtained from the case. Then, her intrauterine device was removed 10 days before the myomectomy, and her beta-human chorionic gonadotropin ( β HCG) test was negative before the operation. An abdominal myomectomy under spinal anesthesia was performed by a 9 cm Pfannenstiel incision of a 10 cm uterine leiomyoma. Upon surgical observation, pelvic organs and peritoneal cavity were normal. A vertical incision was made over the anterior wall of the uterus to remove the intramural fibroid. After the complete removal of the uterine fibroid, the sample was sent to the pathology ward. Additionally, the endometrium was left intact, the necrotic derbies were removed, and the uterine serous was repaired. Based on the pathology examination, the diagnosis of leiomyoma was established.

She was discharged from the hospital after a few days with no complications. Oral contraceptive pills were prescribed to prevent pregnancy for 6 months. One month later, she came back to the clinic due to retarded menstrual cycle. The result of the β HCG test was positive. Subsequently, a single alive fetus was seen in transvaginal ultrasound. According to Crown-rump length measurement, the gestational age was estimated to be 6 wk and 4 days.

Since the woman was a high-risk pregnancy case, close monitoring of fetal growth and maternal health was performed. In the serial ultrasound, the color Doppler of the placenta and the location of the placenta and myomectomy scar (in front of the uterus) were assessed.

**Figure 1:**
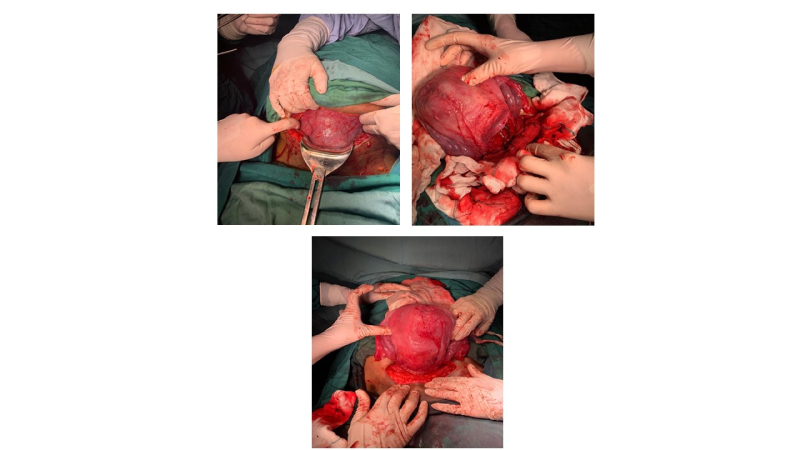
Cesarean section delivery and uterus.

Finally, on the 38 ${\;}^{\text{th}}$ wk of pregnancy, a cesarean section was performed (Figure 1). A healthy girl was born, weighing 3700 gr, with an 8/10 Apgar score. The woman had no complications during pregnancy and cesarean section. Both the healthy mother and newborn were discharged from the hospital 2 days after the cesarean section.

### Ethical considerations

A written informed consent was obtained from the case to use her medical records for any research purpose. Also, the manuscript is written based on the CARE checklist.

## 3. Discussion

Herein, we presented a case of successful term delivery after myomectomy during the first weeks of pregnancy. Based on the ultrasound, at the time of the myomectomy, she was in the luteal phase of the menstrual cycle. Thus, her early pregnancy was not detected in the β HCG. After myomectomy, the woman was advised to prevent pregnancy for at least 6 months. However, about 4 wk after the myomectomy, she presented with retarded menstruation. The pregnancy test was positive, and the gestational age was estimated to be 6 wk and 4 days in the ultrasound and we found that she was pregnant during the surgery.

During pregnancy, most uterine fibroids are asymptomatic and stable in size and cause no considerable complication. However, in rare cases, they may lead to serious circumstances such as spontaneous abortion, placenta previa, fetal malpresentation, placental abruption, and postpartum hemorrhage (1). Some uterine fibroid symptoms including dyspareunia led to avoidance of sexual intercourse that can interfere with fertility (7). On the other hand, the safety of myomectomy during pregnancy remains controversial. It is usually associated with a higher risk of uterine rupture during labor or in the third trimester (6, 8). The most important factors in determining morbidity in pregnancy are size, location, and number of uterine fibroids (9).

Myomectomy during pregnancy is commonly associated with serious complications such as miscarriage, preterm labor, abnormal placentation, hemorrhage, uterine rupture, and potential hysterectomy (4). There is no consensus on the optimal time interval between myomectomy and pregnancy (5). It is recommended to avoid myomectomy and closely monitor uterine fibroid changes during pregnancy (9). However, in cases of symptomatic uterine fibroids with poor response to medications, myomectomy can be considered (10).

Some studies reported successful myomectomy during pregnancy. They recommended this procedure as a safe option in cases with pressure symptoms during pregnancy as a result of enlarged leiomyoma (4, 11). Similar to our case, 2 studies reported successful myomectomy at 6–7 wk of gestational age that resulted in one cesarean section delivery and one vaginal delivery with good outcomes. The uterine fibroid location in the case of vaginal delivery was within the cervix and surgery was done by hysteroscopy through the canal without complication (12, 13). In this case, 3 important factors contributed to safe pregnancy. First, a fibroid was located within the anterior wall of the uterus. Second, placenta implantation was in the posterior wall of the uterus. Third, the endometrium was intact. A previous study also reported that these factors might lead to a lower risk of further complications during pregnancy (4).

The mean time interval between myomectomy and pregnancy was reported 17.6 ± 9.2 months in a review. However, the optimal time interval is unclear. Based on clinical manifestations, the interval between myomectomy and pregnancy could be shortened to 3 months. Since uterus rupture is the most serious complication after myomectomy, it is important to make the women aware about the signs (acute abdominal pain, bleeding). This complication mostly happens in the third trimester of pregnancy and no correlation was found with the interval between myomectomy and pregnancy and the time of occurrence of spontaneous uterine rupture during pregnancy. There is a hypothesis that scar healing of myomectomy could happen during early pregnancy because of the small growth of the uterus so there could be no minimal interval between myomectomy and getting pregnant (5).

## 4. Conclusion

In conclusion, symptomatic uterine fibroids myomectomy during early pregnancy can be a safe therapeutic option. Especially, when the placenta and leiomyoma are located in different walls of the uterus, myomectomy procedures can be performed with fewer complications.

##  Data availability

Data supporting the findings of this study are available upon reasonable request from the corresponding author.

##  Author contributions

N. Saharkhiz: Diagnosis, and writing original draft, M. Nemati: Writing, review and editing, N. Hajizadeh: Reviewing the diagnostic findings and editing the draft, H. Abbasi: Supervision. All authors approved the final manuscript and take responsibility for the integrity of the data.

##  Conflict of Interest

The authors declare no conflict of interest.
